# Malignant erythema as a high-specificity cutaneous indicator of malignancy in adult dermatomyositis: a retrospective cohort study

**DOI:** 10.3389/fimmu.2026.1960856

**Published:** 2026-09-30

**Authors:** Xiaojing Xing, Zihang Li, Biwen Lin

**Affiliations:** 1 Department of Dermatology, Capital Medical University Affiliated Beijing Shijitan Hospital, Beijing, China; 2 Department of Dermatology, First Medical Centre of Chinese People's Liberation Army (PLA) General Hospital, Beijing, China

**Keywords:** anti-TIF-1 γ antibody, cancer risk, cutaneous biomarkers, dermatomyositis, malignant erythema

## Abstract

**Background:**

Dermatomyositis (DM) is associated with a substantially elevated risk of malignancy, yet the cutaneous features predictive of cancer risk in adults remain insufficiently defined.

**Objectives:**

This study aimed to characterize cutaneous manifestations and clinical factors associated with malignancy in adult DM, with a focus on malignant erythema.

**Methods:**

This retrospective cohort study included 261 adults with DM admitted between 2016 and 2023 and followed up till June 2025. Clinical data, laboratory indicators (including creatine kinase [CK] and lactate dehydrogenase [LDH]), autoantibodies, and skin findings were systematically evaluated. The primary outcome was the occurrence of malignant tumors.

**Results:**

Twenty‑six patients (10%) developed malignancy. Malignant erythema was identified in 35 patients (13.3%), among whom 14 (40%) had malignancy. Both anti-transcription intermediary factor-1 γ (anti-TIF-1 γ) antibodies and malignant erythema were independently associated with malignancy (odds ratio [OR] = 18.231, 95% confidence interval [CI] 4.568–72.756, P < 0.001; OR = 17.588, 95% CI 2.151–143.824, P = 0.007). Malignant erythema demonstrated higher specificity for tumor prediction than anti-TIF-1 γ antibodies (91.1% vs. 86%, P < 0.001), while sensitivity did not differ significantly (53.8% vs. 73.1%, P = 0.190). while.

**Conclusion:**

Malignant erythema is a distinctive poikilodermatous manifestation strongly associated with malignancy in adult DM. Its high specificity suggests that malignant erythema may serve as a valuable clinical indicator for malignancy risk and a useful complement to anti-TIF-1 γ antibody testing.

## Introduction

Dermatomyositis (DM) is a subtype of idiopathic inflammatory myopathy (IIM) characterized by proximal muscle weakness and distinctive cutaneous lesions (1, 2). Unlike many other connective tissue diseases, adult-onset DM carries a substantial risk of malignancy, and cancer has been recognized as a major contributor to morbidity and mortality in this population (3–6). Epidemiological evidence consistently demonstrates that malignancy is a major comorbidity in DM. Contemporary cohort studies and recent systematic reviews report that approximately 10–25% of patients with DM develop cancer (7), and the standardized incidence ratio (SIR) remains markedly elevated—typically between 2.4 and 6.2—compared with the general population (8). Despite this well-established association, not all patients with DM will develop cancer, and accurately identifying those at highest risk remains a critical clinical challenge (8–10).

As a result, considerable interest has focused on serological biomarkers that may aid in malignancy risk stratification (11). Among them, anti–transcription intermediary factor-1 γ (anti-TIF-1 γ) antibodies are the most consistently reported markers associated with cancer‑associated DM in adults (12, 13). In addition to anti-TIF-1 γ antibodies, NXP-2 antibodies have been reported to be associated with an increased risk of malignancy in certain populations, although this association appears less consistent across cohorts (14, 15). However, the clinical characteristics and cutaneous phenotypes of adult DM patients who develop malignancy have not been comprehensively defined. In particular, there is a lack of in-depth studies integrating detailed skin manifestations with serological profiles to refine malignancy risk stratification in DM.

Previous work has suggested that, in patients with DM, the presence of malignant tumors is clinically associated with a high prevalence of poikiloderma on the forehead and upper back, and with more extensive skin involvement (16). Early Chinese dermatology textbooks describe a special DM skin manifestation termed malignant erythema, considered a particular form of poikiloderma. These lesions are fire-red, wine-red, or brownish-red, with pigmentation, telangiectasia, occasional bran-like scales, and sometimes poikiloderma, and are widely distributed on the scalp, face, neck, upper trunk, and occasionally proximal extremities (17). Chinese dermatologists have long proposed that malignant erythema represents a distinct poikilodermatous pattern in DM and may be associated with an increased risk of malignant tumors (18).

In the present study, we aimed to systematically characterize the cutaneous manifestations and clinical features of adult DM patients with malignant tumors and to identify factors—particularly malignant erythema and anti-TIF-1 γ antibodies—associated with malignancy.

## Materials and methods

### Study design and patient selection

This single‑center retrospective observational cohort study included all consecutive adult patients diagnosed with dermatomyositis (DM) who were admitted to the inpatient dermatology department of the First Medical Center of the People’s Liberation Army (PLA) General Hospital between 1 March 2016 and 30 September 2023, with follow‑up continued until 30 June 2025. Eligible patients were required to be ≥18 years of age at disease onset, meet the 2017 EULAR/ACR classification criteria for definite idiopathic inflammatory myopathies (1), and have complete baseline clinical records, laboratory data, cutaneous assessments, and follow‑up information sufficient to determine malignancy status and survival outcomes. Patients with inclusion body myositis, polymyositis, overlap myositis, muscular dystrophy, metabolic or drug‑induced myopathy, and patients with a history of malignancy diagnosed prior to or at the time of DM diagnosis, or with incomplete clinical data, were excluded. The study protocol was approved by the Institutional Ethical Standards Committee of the First Medical Center, PLA General Hospital (approval number: S2017-718-12).

### Data collection

All clinical data were extracted from the hospital’s electronic medical record (EMR) system. No data were obtained from external sources. Extracted variables included demographic characteristics, disease onset age, disease duration, follow-up time, clinical symptoms, laboratory indicators, myositis-specific autoantibodies (MSAs), myositis-associated autoantibodies (MAAs), and cutaneous manifestations. Erythema of exposed areas was defined as erythema affecting sun-exposed skin, including the face, neck, V-area of the chest, dorsal aspects of the hands, and extensor surfaces of the forearms.

### Definition of malignant erythema

Malignant erythema was defined according to consensus descriptions in Chinese dermatology textbooks and clinical practice guidelines (17, 18). Although this term does not appear in English-language literature, its clinical features overlap with the severe poikilodermatous erythema described in anti-TIF-1 γ - positive or cancer-associated dermatomyositis (16, 19). It refers to a distinctive poikilodermatous erythema characterized by diffuse fire‑red, wine‑red, or brownish‑red patches with prominent telangiectasia and pigmentary alteration, occasionally accompanied by fine bran‑like scaling, and typically distributed on the scalp, face, neck, upper trunk, and proximal extremities. The diagnosis was established independently by two experienced dermatologists (XX and ZL) based on clinical photographs and detailed physical examination records. In cases of diagnostic disagreement, a third senior dermatologist (BL) adjudicated the final classification. Formal Cohen’s kappa statistics were not calculated retrospectively given the study design but will be incorporated in future prospective validation studies.

### Laboratory indicators and antibody testing

Fasting serum samples were collected at the time of first hospital admission, prior to initiation of systemic immunosuppressive therapy. Samples were processed within 2 hours and stored at −80 °C until analysis. Sixteen MSAs/MAAs—including anti-TIF1-γ, anti-MDA5, anti-Mi-2, anti-NXP2, anti-SAE, anti-Jo-1, anti-PL-7, anti-PL-12, anti-EJ, anti-OJ, anti-SRP, anti-PM-Scl75, anti-PM-Scl100, anti-Ku, and anti-Ro52—were measured using a commercial line-blot immunoassay (EUROLINE Myositis Profile, Euroimmun, Lübeck, Germany) according to the manufacturer’s instructions and repeated for 3 times, and results consistent in no less than two replicates were adopted as the final result. Creatine kinase (CK) and lactate dehydrogenase (LDH) levels were measured using standard automated biochemical analyzers in the hospital’s central laboratory.

### Outcomes and assessments

The primary outcome was the presence of malignant tumors diagnosed by high-resolution chest CT, abdominal and pelvic MRI, gastrointestinal and nasopharyngeal endoscopy, ultrasonography, serum tumor markers, PET-CT, and pathological examination when clinically indicated during follow-up period. Secondary outcomes included interstitial lung disease (ILD) diagnosed by chest CT and death during follow-up period.

### Statistical analyses

SPSS (version 24.0; IBM Corp., Armonk, NY, USA) was used for all the statistical analyses. χ^2^ or Fisher’s exact tests were used for binary data comparisons. Continuous data were expressed as mean ± standard deviation (SD) or median (Q1, Q3), and comparisons were made using Student’s t-test or Mann-Whitney U rank-sum test between two groups. Logistic regression analysis was used to identify independent risk factors for malignancy. First, univariate logistic regression was performed for all clinical, serological and cutaneous variables; variables with P < 0.05 in univariate analysis were selected as candidate covariates and entered into the multivariate model via forced entry method. Variance Inflation Factor (VIF) was calculated to evaluate multicollinearity among covariates; VIF > 5 was defined as moderate multicollinearity and VIF > 10 as severe multicollinearity. The interaction term between anti-TIF-1 γ antibody and malignant erythema was further incorporated to explore their synergistic predictive effect. The age cutoff of 50 years was selected *a priori* based on the median age of malignancy onset in our cohort and consistent with cancer screening recommendations in Asian populations. We acknowledge that the International Myositis Assessment and Clinical Studies Group (IMACS) risk stratification guideline uses an age cutoff of 40 years for enhanced malignancy screening; however, sensitivity analyses using alternative cutoffs did not materially alter the main findings (data not shown). Covariates with P < 0.05 in multivariate analysis were identified as independent predictors of malignancy. Sensitivity and specificity of predictive indicators were compared between the groups using the McNemar test. Prediction efficiency was tested by comparing area under receiver operating characteristic (AUROC) curves using the method described by Hanley and McNeil (MedCalc, version 20) (20). *P* < 0.05was considered statistically significant.

## Results

### Baseline characteristics

To characterize the clinical differences between patients with and without malignancy, we first compared baseline demographic and clinical features. Among the 261 adult inpatients with DM, 26 (10.0%) developed malignant tumors and 235 (90.0%) did not (**Figure 1**) during follow-up. Among the 26 patients who developed malignancy, including lung cancer (n = 5), gastric cancer (n = 4), colorectal cancer (n = 3), breast cancer (n = 3), nasopharyngeal carcinoma (n = 7), and others (n = 4). The median time from DM diagnosis to cancer detection was 12.3 months (interquartile range, 9.8–37.2 months), with 12 patients (46.2%) diagnosed within the first year of DM onset. The sex distribution was similar between groups (P = 0.809). Patients with malignancy had a significantly older age at onset of DM (56.54 ± 15.00 vs. 47.70 ± 12.89 years, P = 0.001) and a shorter disease course at first admission (P = 0.001), whereas follow‑up duration did not differ significantly (P = 0.268).

**Figure 1:**
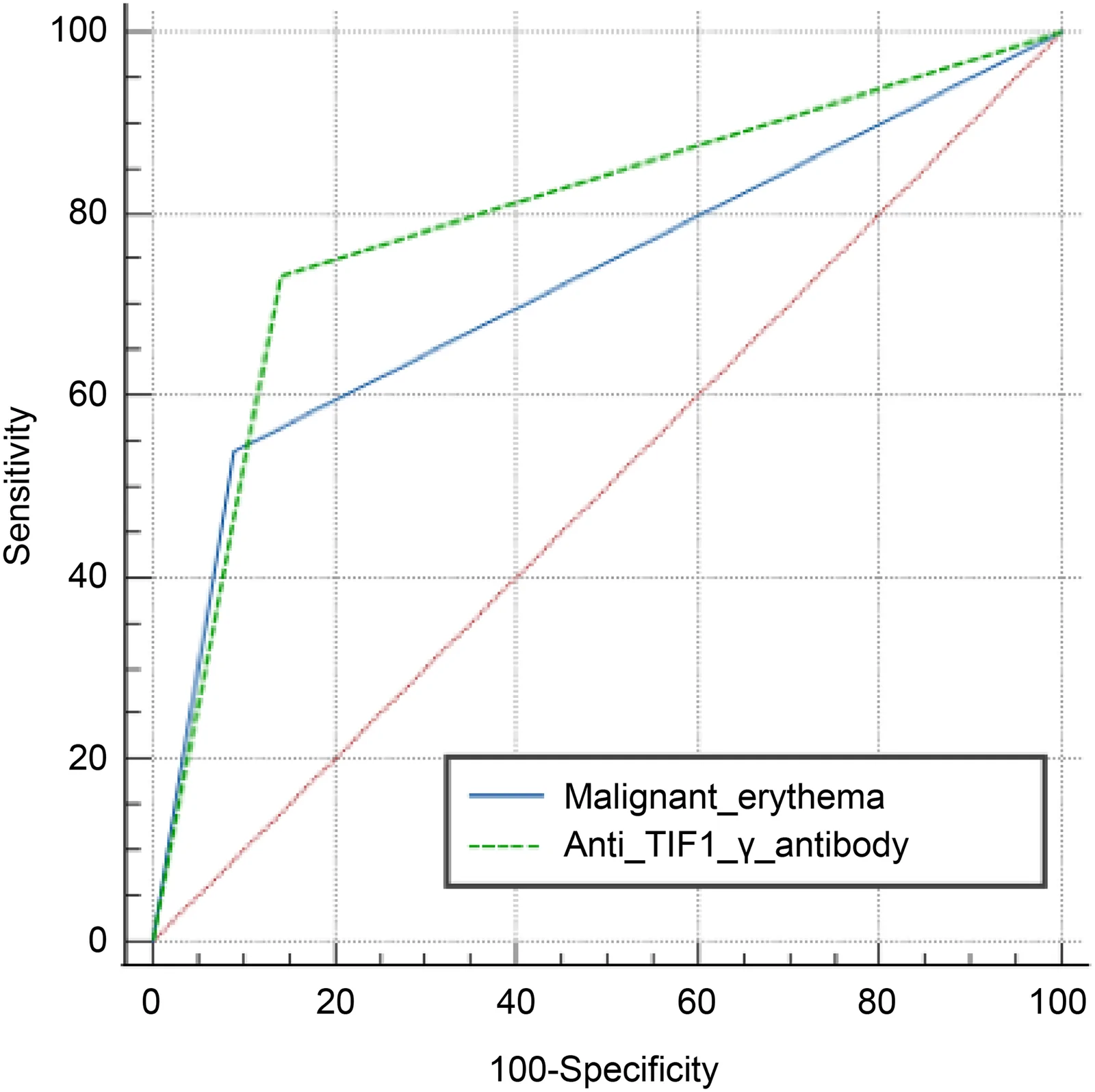
Patients inclusion flowchart. DM, dermatomyositis.

We next examined systemic symptoms and laboratory findings. Myalgia, arthralgia and arthritis showed no significant differences between groups (P = 0.068 and P = 0.557, respectively). Dysphagia was more frequent in the malignancy group (53.8% vs. 31.1%, P = 0.019). Elevated CK and LDH levels were comparable (P = 0.750 and P = 0.400, respectively). The prevalence of ILD did not differ significantly (P = 0.219). Mortality was markedly higher in the malignancy group (30.8% vs. 3.8%, P < 0.001). Anti-TIF-1 γ antibody positivity was strongly associated with malignancy (73.1% vs. 14.0%, P < 0.001) (**Table 1**). NXP-2 antibody positivity showed no significant association with malignancy in this cohort (7.7% in the malignancy group vs. 11.1% in the non-malignancy group, P = 0.847) (**Table 1**). Furthermore, among the 35 patients with malignant erythema, only two (5.7%) were positive for anti-NXP-2 antibodies, precluding a meaningful assessment of its relationship with this specific cutaneous phenotype.

**Table 1:** Characteristics of 261 patients with dermatomyositis and malignant tumors.

Patient characteristic	Malignant tumor		P-value
	Positive (n = 26)	Negative (n = 235)	
Male/female	8/18	67/168	0.809
Age at onset, mean (SD), years	56.54 ± 15.00	47.70 ± 12.89	0.001
Course of disease (median, Q), months^*^	3 (3, 10)	8 (3, 24)	0.001
Follow-up time (median, Q), years	5.3 (3.0, 4.0)	5.5 (6, 6.5)	0.268
Myalgia (N), n (%)	24/2 (92.3%)	174/61 (74%)	0.068
Arthralgia and arthritis, (N) n (%)	8/18 (30.8%)	86/149 (36.6%)	0.557
Dysphagia (N), n (%)	14/12 (53.8%)	73/162 (31.1%)	0.019
Elevated CK (N), n (%)	10/16 (38.5%)	98/137 (41.7%)	0.750
Elevated LDH (N), n (%)	20/6 (76.9%)	162/73 (68.9%)	0.400
ILD (N), n (%)	12/14 (46.2%)	138/97 (58.7%)	0.219
Death	8/18 (30.8%)	9/226 (3.8%)	<0.001
Myositis-specific autoantibodies			
Anti-EJ antibody	1/25 (3.8%)	5/230 (2.1%)	1.000
Anti-Jo-1 antibody	2/24 (7.7%)	22/213 (9.4%)	1.000
Anti-MDA-5 antibody	3/23 (11.5%)	74/161(31.5%)	0.059
Anti-Mi-2 antibody	4/22 (18.2%)	34/201 (16.9%)	1.000
Anti-NXP-2 antibody	2/24 (7.7%)	26/209 (11.1%)	0.847
Anti-PL-7 antibody	2/24 (7.7%)	21/214 (8.9%)	1.000
Anti-PL-12 antibody	2/24 (7.7%)	6/229 (2.6%)	0.399
Anti-SRP antibody	1/25 (3.8%)	22/213 (9.4%)	0.564
Anti-TIF1-γ antibody	19/7 (73.1%)	33/202 (14%)	<0.001
Myositis-associated autoantibodies			
Anti-Ku antibody	0/26 (0%)	13/222 (5.5%)	0.375
Anti-PM/Scl75 antibody	1/25 (3.8%)	19/226 (7.8%)	0.741
Anti-PM/Scl100 antibody	0/26 (0%)	10/225 (4.3%)	0.605
Anti-Ro52 antibody	11/15 (42.3%)	126/109 (53.6%)	0.273
Cutaneous manifestations			
Heliotrope rash (N), n (%)	20/6 (76.9%)	125/110 (53.2%)	0.021
Gottron’s sign/Gottron’s papules (N), n (%)	14/12 (53.8%)	150/85 (63.8%)	0.318
Poikiloderma (N), n (%)	6/20 (23.1%)	38/197 (16.2%)	0.372
Holster sign (N), n (%)	9/17 (34.6%)	55/180 (23.4%)	0.207
Erythema of exposure area (N), n (%)	21/5 (80.8%)	158/77 (67.2%)	0.158
Mechanic’s hands (N), n (%)	1/25 (3.8%)	49/186 (20.9%)	0.068
Scalp erythema (N), n (%)	9/17 (34.6%)	14/221 (6.0%)	<0.001
Malignant erythema (N), n (%)	14/12 (53.8%)	21/214 (8.9%)	<0.001

ILD, interstitial lung disease; LDH, lactate dehydrogenase; CK, creatine kinase. ^*^ Mann-Whitney U rank-sum test.

Given the importance of cutaneous phenotypes in DM, we further compared skin manifestations between groups. Heliotrope rash, scalp erythema, and malignant erythema were significantly more common in patients with malignancy (P = 0.021, P < 0.001, and P < 0.001, respectively), whereas Gottron’s sign/papules, poikiloderma, Holster’s sign, erythema of exposed areas, and mechanic’s hands were not significantly associated with malignancy.

### Logistic regression analysis

To identify independent factors associated with malignancy, we performed multivariable logistic regression. Anti-TIF-1 γ antibody positivity (OR = 18.231, 95% CI 4.568–72.756, P < 0.001) and malignant erythema (OR = 17.588, 95% CI 2.151–143.824, P = 0.007) remained significant predictors of malignancy. A significant interaction was observed between anti-TIF-1 γ antibodies and malignant erythema (P = 0.023), suggesting a potential synergistic effect. In contrast, age >50 years, disease course <6 months, dysphagia, heliotrope rash, and scalp erythema were not independently associated with malignancy (**Table 2**).

**Table 2:** Logistic regression analysis to test the influence factors of malignant tumors.

Variables	OR	95% CI	P-value
Age > 50 years	1.785	0.655–4.862	0.257
Course < 6 month	2.163	0.801–5.840	0.128
Dysphagia	1.007	0.361–2.814	0.989
Heliotrope rash	1.477	0.488–4.471	0.490
Scalp erythema	2.066	0.587–7.273	0.259
Anti-TIF-1 γ antibody	18.231	4.568–72.756	<0.001
Malignant erythema	17.588	2.151–143.824	0.007
Anti-TIF-1 γ antibody by malignant erythema	20.758	1.587-160.352	0.023

CI, confidence interval.

### Clinical features of patients with malignant erythema

To further characterize this distinctive cutaneous phenotype, we analyzed the clinical features of the 35 patients (13.3%) with malignant erythema. Their mean age at onset was 52.54 ± 15.85 years, and most were female (68.5%). Anti-TIF-1 γ antibody positivity was observed in 30 patients (85.7%). Malignant erythema predominantly involved the face (100%), neck (85.7%), chest (74.3%), back (80%), and proximal upper extremities (88.6%), including the upper arms, forearms, and shoulders. Representative clinical images are shown in **Figure 2**; **Supplementary Figure 1**.

**Figure 2:**
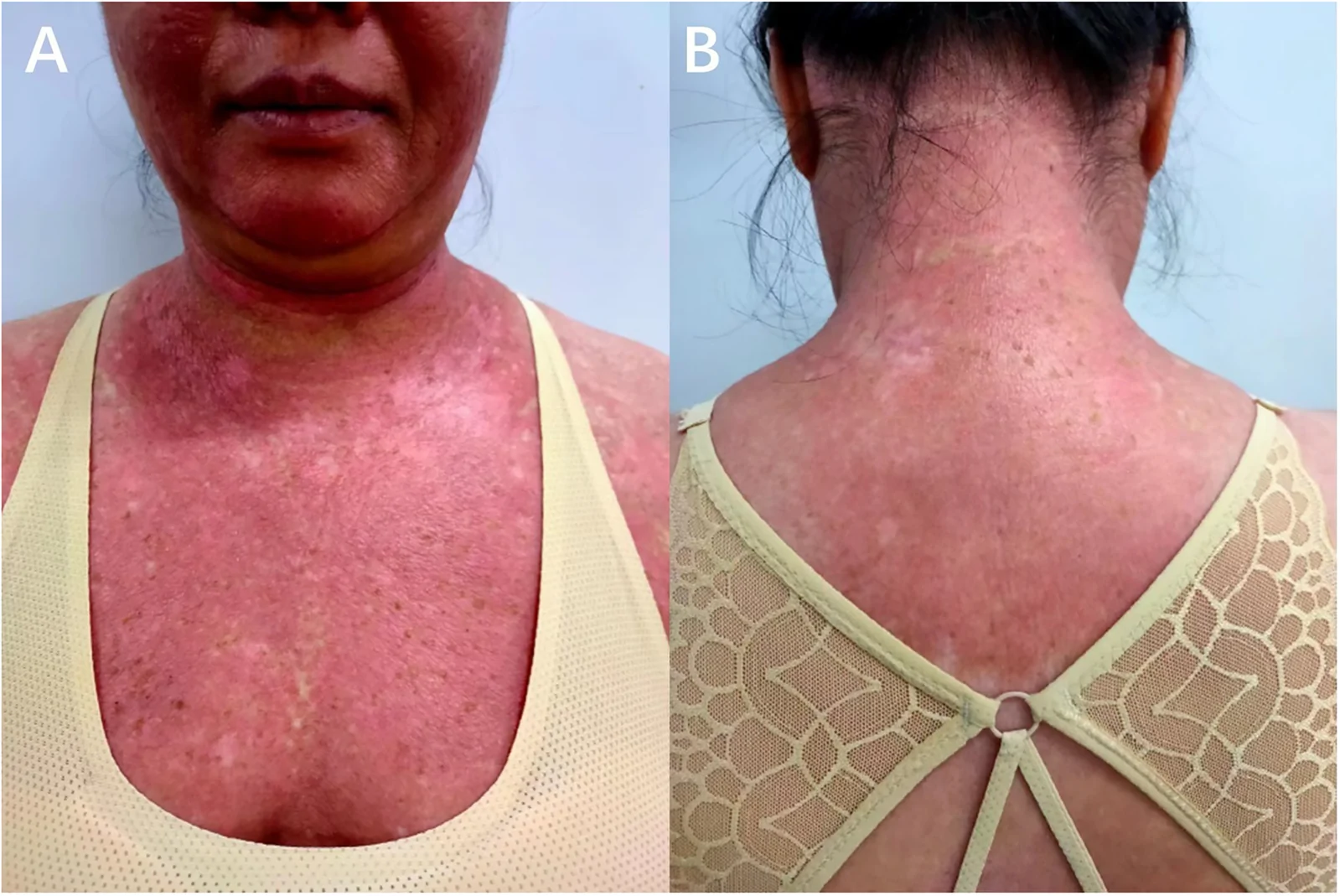
**(a)** Malignant erythema distributed across the face, neck, and shawl; **(b)** scalp, neck, shawl.

We then evaluated clinical outcomes in this subgroup. Of the 35 patients with malignant erythema, 14 (40%) developed malignancy, 10 (28.6%) had ILD, and 6 (17.1%) died—all due to malignant tumors. Coexisting cutaneous features included erythema of exposed areas (91.4%), heliotrope rash (80%), scalp erythema (40%), poikiloderma (51.4%), and Holster’s sign (54.3%) (**Table 3**).

**Table 3:** Clinical features of patients with malignant erythema (N = 35).

Variables	Results (%)
Age at onset	52.54 ± 15.85
Sex	
Male	11(31.4)
Female	24 (68.5)
Dysphagia	19 (54.3)
Myalgia	32 (91.4)
Arthralgia and arthritis	7 (20.0)
Myositis antibodies	
Anti-TIF1-γ antibody	30 (85.7)
Anti-PM/Scl75 antibody	2 (5.7)
Anti-SAE-1 antibody	1 (2.9)
Negative	2 (5.7)
Location	
Scalp	16 (45.7)
Face	35 (100)
Neck	30 (85.7)
Chest	26 (74.3)
Abdomen	10 (28.6)
Back	28 (80.0)
Lateral thigh	16 (45.7)
Upper extremities (including upper arms and forearms)	31 (88.6)
Lower limbs	24 (70.8)
Malignant tumor	
Yes	14 (40)
No	21 (60)
ILD	
Yes	10 (28.6)
No	25 (71.4)
Death	6 (17.1)
Heliotrope rash	28 (80.0)
Erythema of the exposure area	32 (91.4)
Poikiloderma	18 (51.4)
Holster sign	19 (54.3)
Scalp erythema	14 (40.0)

ILD, interstitial lung disease.

### Predictive performance of malignant erythema and anti-TIF-1 γ antibodies

To compare the predictive value of malignant erythema and anti-TIF-1 γ antibodies, we assessed their sensitivity, specificity, and AUROC. Malignant erythema demonstrated higher specificity for tumor prediction than anti-TIF-1 γ antibodies (91.1% vs. 86%, P < 0.01), while sensitivity did not differ significantly (73.1% vs. 53.8%, P = 0.190) (**Supplementary Table 1**). The AUROC for anti-TIF-1 γ antibodies was 0.795 (95% CI, 0.692–0.898), compared with 0.725 (95% CI, 0.604–0.845) for malignant erythema, with no significant difference between the two (P = 0.249) (**Figure 3**). These findings indicate that malignant erythema may serve as a clinically useful, highly specific indicator of malignancy risk.

**Figure 3:**
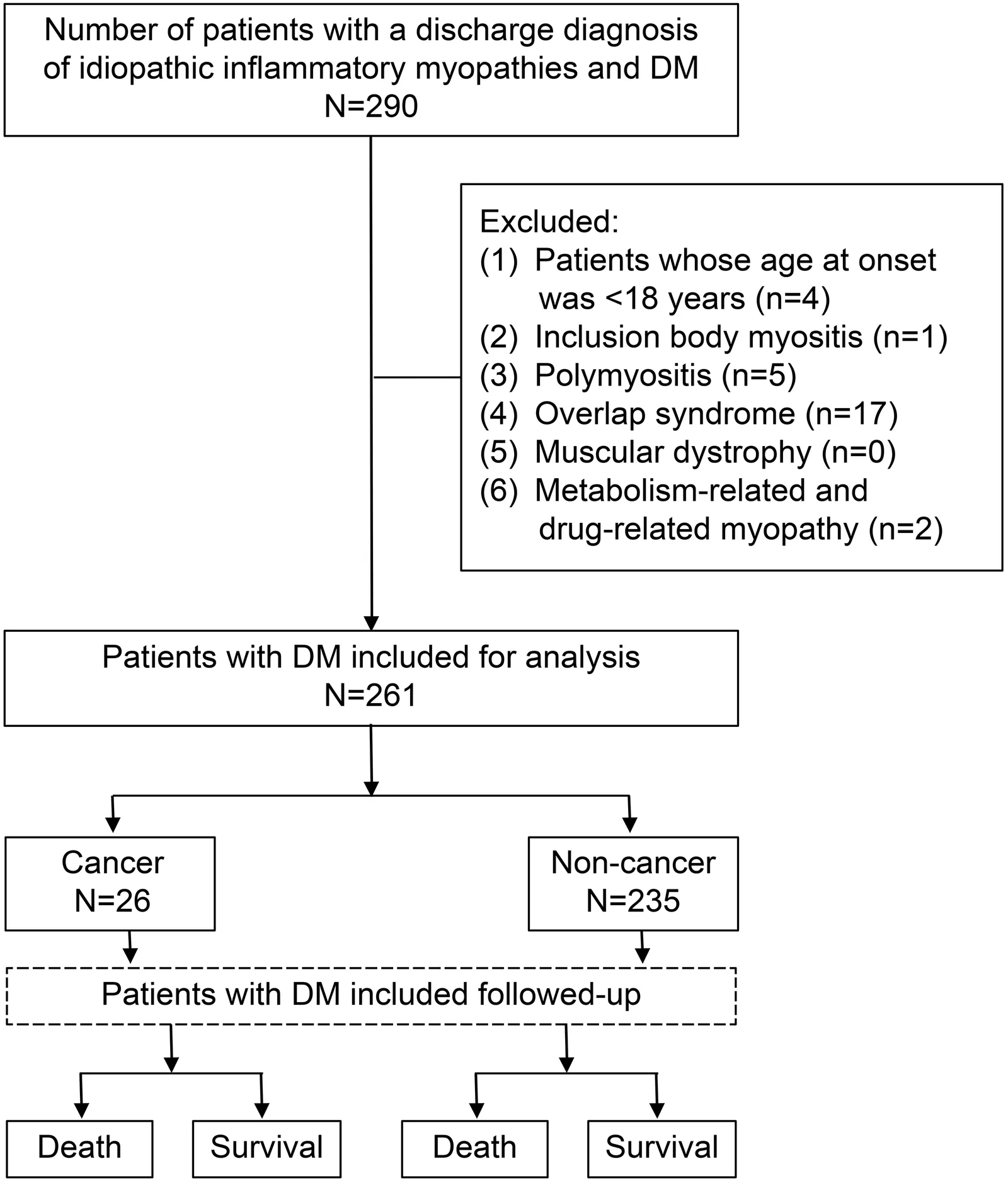
Predictive performance of malignant erythema and Anti-TIF-1 γ antibodies for cancer during follow-up. Area under the receiver operating characteristic curve (AUROC) values are presented with 95% confidence intervals: Anti-TIF-1 γ antibodies, 0.795 (95% CI, 0.692–0.898); malignant erythema, 0.725 (95% CI, 0.604–0.845).

## Discussion

This retrospective cohort study identified malignant erythema as a distinctive cutaneous phenotype strongly associated with malignancy in adult DM. Among 261 patients, 10% developed malignant tumors, and malignant erythema was present in 13.3% of the cohort, with 40% of these patients subsequently diagnosed with cancer. Both malignant erythema and anti-TIF-1 γ antibodies emerged as independent malignancy-associated factors, highlighting their potential value in risk stratification.

Although our study did not calculate a standardized incidence ratio (SIR), the 10% malignancy rate observed in our cohort is consistent with previous epidemiological studies reporting markedly elevated cancer risk in DM, with SIR values ranging from 2.4 to 6.2 in different populations (7, 8). Similar to earlier reports, patients with malignancy in our cohort were older at disease onset and had a shorter disease course at presentation, suggesting a more abrupt and aggressive clinical phenotype (7). The strong association between anti-TIF-1 γ antibodies and malignancy observed in our study aligns with prior evidence identifying anti-TIF-1 γ antibodies as one of the most reliable serological markers for cancer-associated DM (12, 13). However, unlike some cohorts in which ILD was inversely associated with malignancy (21–23), the prevalence of ILD did not differ significantly between groups in our study, possibly reflecting differences in ILD severity, population characteristics, or referral patterns.

The close association between malignant erythema and malignancy raises important mechanistic considerations. Malignant erythema is characterized by extensive, intensely erythematous poikiloderma with telangiectasia and pigmentary alteration, often involving the face, neck, and upper trunk. Its high co-occurrence with anti-TIF-1 γ antibodies (85.7% in our cohort) suggests that malignant erythema may represent a cutaneous phenotype driven by tumor-related immune responses. Prior studies have demonstrated that patients with anti-TIF-1 γ antibodies consistently exhibit upregulation of anti-TIF-1 γ expression in both muscle and skin tissues, which may be associated with enhanced autoimmune responses and contribute to severe cutaneous inflammation (19). This immunopathologic pattern may explain why malignant erythema is both clinically distinctive and closely linked to malignancy. Although anti-SAE antibodies have been associated with severe and diffuse cutaneous erythema in previous reports (24, 25), only one patient (2.9%) in our malignant erythema subgroup tested positive for anti-SAE antibodies, precluding a meaningful analysis of this association in the present cohort.

The clinical implications of these findings are notable. Malignant erythema is readily identifiable during routine dermatologic examination and may serve as a practical, non-invasive indicator of malignancy risk in DM. Compared with anti-TIF-1 γ antibodies, malignant erythema demonstrated higher tumor prediction specificity, suggesting that its presence may help refine cancer screening strategies, particularly in settings where serological testing is limited. Incorporating malignant erythema into malignancy risk assessment models may improve early tumor detection and guide individualized surveillance, consistent with recent calls for risk-based cancer screening in DM (7). Although general poikiloderma was not independently associated with malignancy in our cohort, malignant erythema represents a distinct, severe poikilodermatous phenotype characterized by intensely fire-red or wine-red erythema, prominent telangiectasia, marked pigmentary alteration, and extensive distribution involving the scalp, face, neck, and upper trunk. These specific morphological and distributional features distinguish malignant erythema from nonspecific poikiloderma, suggesting that not all poikilodermatous changes carry equivalent malignant risk.

This study has several strengths, including a relatively large single-center cohort, standardized assessment of cutaneous manifestations, and comprehensive malignancy screening. However, several limitations should be acknowledged. First, the number of patients with malignancy was modest, which may limit the statistical power of subgroup analyses. Second, the retrospective design may introduce selection bias toward more severe systemic phenotypes, including a higher prevalence of ILD among those with malignancy. Consequently, the predictive value of malignant erythema in dermatology outpatients presenting with predominant skin disease remains to be validated in prospective, community-based studies. Furthermore, this retrospective study did not systematically collect Cutaneous Dermatomyositis Disease Area and Severity Index (CDASI) scores; therefore, we were unable to evaluate the correlation between quantitative skin disease activity and malignancy risk. Prospective studies incorporating CDASI are warranted to refine cutaneous risk stratification. Third, detailed radiological patterns of interstitial lung disease (e.g., NSIP vs. UIP) and their specific association with lung cancer were not systematically analyzed in this retrospective cohort. Additionally, the relationship between anti-Ro52 positivity and malignancy among patients with coexisting ILD could not be fully elucidated due to the limited number of events. These represent important directions for future investigation. In addition, the limited number of patients with malignant tumors (n=26) may reduce the statistical stability of multivariate logistic regression and subgroup analyses, even though we strictly controlled multicollinearity and reported 95% CIs for all odds ratios to quantify estimation uncertainty. Fourth, given the modest number of malignancy events (n = 26), the AUROC estimates and their direct comparison should be interpreted with caution. The non-significant difference in AUC between anti-TIF-1 γ antibodies and malignant erythema (P = 0.249) may reflect insufficient statistical power rather than true equivalence between the two predictors. Bootstrap internal validation with optimism-corrected AUC values was not performed due to the limited event number and retrospective design but is recommended for future larger, prospective studies to confirm these findings. Fifth, as a single-center study conducted at a tertiary referral hospital, our cohort may have been enriched with more severe or complicated cases, potentially overestimating the prevalence of malignant erythema and malignancy compared with outpatient or community-based populations. This limits the generalizability of our findings to patients with milder disease presenting in dermatology clinics or primary care settings. Finally, as a single-center study based on a Chinese cohort, the generalizability of the findings to other populations may be limited.

## Conclusion

Malignant erythema is a distinctive poikilodermatous manifestation in adult DM and is strongly associated with malignancy. Its high specificity for malignancy prediction suggests that malignant erythema may serve as a valuable clinical indicator of cancer risk and a useful complement to anti-TIF-1 γ antibody testing. Future multicenter studies with larger cohorts and prospective designs are needed to validate these findings and further elucidate the underlying mechanisms.

## Data Availability

The original contributions presented in the study are included in the article/**Supplementary Material**. Further inquiries can be directed to the corresponding author.
