# Supplementary material for: Malignant erythema as a high-specificity cutaneous indicator of malignancy in adult dermatomyositis: a retrospective cohort study
Source: Front Immunol. 2026 Sep 30;17:1960856. doi: 10.3389/fimmu.2026.1960856 (PMC13661934; doi:10.3389/fimmu.2026.1960856)
Supplement: Supplementary file 1 [file Supplementaryfile1.docx]

**Supplementary Table 1. Comparison of the sensitivity and specificity of malignant erythema and anti-TIF1-𝛾 antibodies in predicting tumours (n=261)**

|  | Malignant tumours | | Predicting Cancer | |
| --- | --- | --- | --- | --- |
| Classified Group | **Cancer** | **Non-cancer** | **Sensitivity (95% CI)** | **Specificity (95% CI)** |
| Anti-TIF1-𝛾 antibody | | | | |
| -Positive | 19 | 33 | 0.731(0.519-0.876) | 0.860(0.807-0.900) |
| -Negative | 7 | 202 |  |  |
| Malignant erythema | | | | |
| -Yes | 14 | 21 | 0.538(0.334-0.728) | 0.911(0.864-0.942) |
| -No | 12 | 214 |  |  |
| P-value |  |  | 0.190 | **0.000** |

**Supplementary Figure 1**. (a-b) back, buttocks, and upper limbs

The malignant erythema is diffuse with wine-red coloration and is accompanied by pigmentation, hypopigmentation, and telangiectasia.

 a b
